# DNA methylation data by sequencing: experimental approaches and recommendations for tools and pipelines for data analysis

**DOI:** 10.1186/s13148-019-0795-x

**Published:** 2019-12-12

**Authors:** Ieva Rauluseviciute, Finn Drabløs, Morten Beck Rye

**Affiliations:** 1 Department of Clinical and Molecular Medicine, NTNU - Norwegian University of Science and Technology, P.O. Box 8905, NO-7491 Trondheim, Norway; 2 Clinic of Surgery, St. Olavs Hospital, Trondheim University Hospital, NO-7030 Trondheim, Norway

**Keywords:** DNA methylation, Hydroxymethylation, Bisulfite sequencing, Computational pipelines

## Abstract

Sequencing technologies have changed not only our approaches to classical genetics, but also the field of epigenetics. Specific methods allow scientists to identify novel genome-wide epigenetic patterns of DNA methylation down to single-nucleotide resolution. DNA methylation is the most researched epigenetic mark involved in various processes in the human cell, including gene regulation and development of diseases, such as cancer. Increasing numbers of DNA methylation sequencing datasets from human genome are produced using various platforms—from methylated DNA precipitation to the whole genome bisulfite sequencing. Many of those datasets are fully accessible for repeated analyses. Sequencing experiments have become routine in laboratories around the world, while analysis of outcoming data is still a challenge among the majority of scientists, since in many cases it requires advanced computational skills. Even though various tools are being created and published, guidelines for their selection are often not clear, especially to non-bioinformaticians with limited experience in computational analyses. Separate tools are often used for individual steps in the analysis, and these can be challenging to manage and integrate. However, in some instances, tools are combined into pipelines that are capable to complete all the essential steps to achieve the result. In the case of DNA methylation sequencing analysis, the goal of such pipeline is to map sequencing reads, calculate methylation levels, and distinguish differentially methylated positions and/or regions. The objective of this review is to describe basic principles and steps in the analysis of DNA methylation sequencing data that in particular have been used for mammalian genomes, and more importantly to present and discuss the most pronounced computational pipelines that can be used to analyze such data. We aim to provide a good starting point for scientists with limited experience in computational analyses of DNA methylation and hydroxymethylation data, and recommend a few tools that are powerful, but still easy enough to use for their own data analysis.

## Background

All scientists working with genomic data today encounter a data-rich environment, where computational analysis is becoming a necessity [1]. Big data from experiments is produced, published, and in most cases made freely available in databases to anyone at any time. However, experimental biologists are often not able to analyze these data themselves. Limited computational competence is not the only explanation. The variety of tools for genomic data analysis can be overwhelming, without sufficiently clear guidelines for choosing between different tools and pipelines. Currently available reviews tend to only mention the variety of tools that could be used but without discussing them in more detail [2–5]. Here we try to rectify this situation by providing an overview of currently available tools and pipelines for a specific subset of genomic data, which for this review is DNA methylation sequencing data derived by the most widely used experimental approaches.

DNA methylation is an important feature of the cell and is involved in many biological processes, including transcription regulation, X chromosome inactivation, genomic imprinting, transposon inactivation, embryonic development, and chromatin structure modification [2]. It is also known that DNA methylation patterns are altered in many diseases, including cancer, which makes this epigenetic mark an attractive target for various studies. High-throughput DNA methylation analysis has now become routine in laboratories worldwide. Experimental methods for DNA methylation detection include restriction enzyme-based, affinity enrichment-based, and bisulfite conversion-based approaches [2–8]. Detection of 5-methylcytosine (5mC) can be performed genome-wide or in selected regions, and at different resolutions all the way down to single nucleotide level. After preparing the DNA by one of three abovementioned protocols, DNA is sequenced. Genome-wide detection of 5mC by bisulfite sequencing is regarded as the current gold standard for DNA methylation detection [5, 7, 9, 10].

5mC is the most abundant and most researched epigenetic mark and has been shown to be essential for gene regulation [11, 12]. In addition, a modification associated with DNA demethylation—5-hydroxymethylcytosine (5hmC)—has also been shown to be involved in normal development as well as various diseases [11–13]. Methylated DNA precipitation approach, originally developed for DNA methylation detection, is now adapted to detect 5hmC, while other techniques, such as DNA hydroxymethylation sequencing, are created specifically for 5hmC detection [6].

The computational analysis of DNA methylation sequencing data generated by different experimental approaches can be a challenging task, especially for the scientists with limited experience in this type of data processing. They often want to make use of publicly available datasets to validate their hypotheses or process their own DNA methylation data but can get overwhelmed by the selection of tools and pipelines. To interpret DNA methylation, data must be processed, visualized, and statistically analyzed [2, 3, 5, 7]. The outcome of this workflow should be methylation levels for different positions and regions in the genome, or if two conditions are being compared, differentially methylated positions (DMPs) or regions (DMRs) [2, 3, 7]. One typical example of differential analysis is DNA methylation comparisons between cancer and normal tissues.

Several computational tools have been created for the various experimental approaches, and for individual steps in the data analysis workflow. For a non-expert user, it can be difficult to choose the best tool, or to combine the right tools into a pipeline. As far as we know, there is currently no review paper that discusses and later recommends a few convenient pipelines available for the scientist who wants to get started with DNA methylation data analysis, but who has limited experience in this area. Universal pipelines that can handle all types of DNA methylation and hydroxymethylation sequencing data have yet to be developed. Before this is achieved, there is a need to develop better guidelines for choosing the right tools and pipelines when analyzing DNA methylation and hydroxymethylation sequencing data.

## DNA methylation and hydroxymethylation

DNA methylation is a covalent modification of cytosine nucleotides, usually located in a CpG dinucleotide [14]. DNA is methylated by transferring a methyl group from the donor S-adenosyl-L-methionine (SAM) to the 5′carbon atom of a cytosine, creating 5mC [14–17]. The chemical reaction is implemented by a group of special proteins, termed DNA methyltransferases (DNMTs) [14–16]. In mammals, DNMT3A and DNMT3B are responsible for de novo DNA methylation, while DNMT1 copies methylation patterns during DNA replication [14, 16, 18]. Methyl groups can be removed by ten-eleven translocation (TET) family proteins that include TET1, TET2, and TET3 [14]. Methylation marks in the cell also need to be read, which is done by protein factors such as MeCP2, which has a methyl-binding domain (MBD), but also by other less known proteins that do not have MBDs [14, 19, 20].

DNA methylation in vertebrate genomes occurs mostly in the context of CpG dinucleotides, which often form clusters of different sizes. Regions with high CpG density are defined as CpG islands (CGIs). CGIs are between 300 and 3000 bp long (average of 1000 bp) with greater than 50–55% GC content and observed/expected ratio of CpG to GpC greater than 0.6, although the specific CGI definition often depends on the source [5, 21, 22]. Around 30,000 CGIs have been identified in the human genome [23]. Most promoters (around 70% of annotated genes) are associated with a CGI [21] and they remain largely unmethylated in normal cells [23]. On the other hand, CGIs located in intragenic regions are more often methylated and these regions remain inactive [23].

5hmC is produced by the oxidation of 5mC catalyzed by TET proteins [11–13, 24]. Three proteins TET1, TET2, and TET3 all convert 5mC to 5hmC, while also being able to catalyze further oxidation into 5-formylcytosine (5fC) and 5-carboxylcytosine (5caC) [13, 24]. TET1–3 are 2-oxoglutarate and Fe (II)-dependent dioxygenases and convert 5mC into 5hmC using the co-substrate α-ketoglutarate [12, 24]. 5hmC levels vary between cell types, with higher frequencies in ES cells and the nervous system. Total levels of 5hmC are 14-fold lower compared with 5mC [11, 12]. 5hmC in ES cells is found at transcription start sites (TSSs), gene bodies, and cis-regulatory elements [12].

## Experimental approaches for detecting DNA methylation and hydroxymethylation

There are three main groups of experimental techniques used for genome-wide DNA methylation and hydroxymethylation detection and methylation data production: (1) restriction enzyme-based, (2) affinity enrichment-based, and (3) bisulfite conversion-based methods [2, 3, 5–8]. These three groups describe the mechanism, i.e., how methylated cytosine is recognized in order to differentiate methylated and unmethylated DNA (or hydroxymethylated and non-hydroxymethylated DNA). Bisulfite conversion-based methods are arguably the most commonly chosen approach today [5]. However, for a given study, the most appropriate approach should be chosen according to the specific biological problem being addressed, the quantitative nature and resolution required by the study, and the cost that can be afforded [2].

Two main types of technologies used to detect methylation signals are methylation arrays and sequencing [6]. Before the era of high-throughput sequencing, methylation arrays, such as Illumina Infinium, were widely used to detect methylation signals [2, 3]. Arrays are still relevant today, mostly because they are simple to analyze and provide a sensitivity and specificity that cannot currently be achieved by sequencing methods at a similar cost [25]. In many cases, methylation arrays are sufficient to detect relevant methylation changes in the studies of biological system, for example, they are widely used for cancer methylomes [2]. Furthermore, their reproducibility makes it possible to compare new and previous results [26]. In this context, the transition from array to sequencing can be challenging. However, the resolution of genome-wide methylation offered by sequencing methods provides the possibility to explore methylation patterns far beyond the single-site methylations shown by arrays [2]. Sequencing is thus replacing arrays as the method of choice for methylation profiling, even though the data are more complicated to analyze [3]. Sequencing-based technologies have been developed based on all three groups of experimental techniques mentioned above.

### Restriction enzyme-based approaches

There are specific proteins that can recognize and cleave only unmethylated sequences, leaving methylated DNA intact [2, 6]. *Msp*I, *Hpa*II, *Not*I, *Sma*I, and *Bst*UI are methylation-sensitive restriction enzymes (MREs) and are used as a basis for restriction enzyme-based methods [2]. In MRE-seq, DNA fragments are size-selected (between 40 and 220 bp) and sequenced, and the locations of unmethylated CpG sites are determined [6]. By using this approach, relative DNA methylation levels can be estimated using read coverage. MRE-seq is cost-effective and easy to perform [2]. However, this technique has relatively low coverage, because the method depends entirely on the location of restriction sites [2, 6].

### Affinity enrichment-based approaches

Proteins with MBD or antibodies against 5mC are used to recognize methylated DNA in affinity enrichment approaches [6]. A methylcytosine-specific antibody is used in MeDIP to immunoprecipitate methylated DNA and the fractions are later evaluated by high-throughput sequencing (MeDIP-seq) [2, 27]. The resolution of this technique is 100–300 bp, 1× coverage covers up to 70% of all CpGs in human genome [27]. MeDIP-seq estimates the relative enrichment of methylated DNA across the genome [2, 27]. One important drawback of this approach is a challenge of computational analysis—CpG-rich fragments are more likely to be enriched compared with the regions that are poor in CpG (< 1.5%), and such regions can be underrepresented or interpreted as unmethylated [2, 27]. Therefore, computational corrections are necessary to normalize CpG content. MeDIP-seq can be adapted to hydroxymethylation by choosing an antibody specific to 5hmC (hMeDIP-seq) [2, 11, 24, 28]. The approaches that use different MBD-containing proteins are similarly low-performing in CpG-poor regions, but methyl-capture sequencing (MethylCap-seq) has been shown to cover more regions, while MBD-capture sequencing (MBDCap-seq) is able to detect two times more DMRs compared with MeDIP-seq [6, 8].

### Bisulfite conversion-based approaches

The most popular way to distinguish methylated cytosine from unmethylated is by treating DNA with sodium bisulfite, which deaminates unmethylated cytosine to uracil, while 5mC remains intact [2, 5–7]. After the conversion, uracil is converted to thymine in the PCR step of the protocol. With this strategy, it is possible to study genome-wide methylation patterns. Whole-genome bisulfite sequencing (WGBS, BS-seq) and reduced-representation bisulfite sequencing (RRBS) integrate bisulfite conversion and high-throughput sequencing [2, 7]. The steps of the WGBS are as follows: (1) genomic DNA purification and sonication, (2) end reparation, A-tailing, and methylated adapters ligation, (3) size selection, (4) bisulfite conversion, (5) PCR, and (6) sequencing of the resulting library [2]. WGBS is a standard profiling strategy for example in NIH Roadmap, ENCODE (initially in 2012 RRBS protocol was used [29]), Blueprint, and IHEC projects. By theoretically covering 100% of the cytosine residues in the genome, WGBS is the most informative and accurate method, and is often used to investigate regions outside of CGIs [2, 30]. A major advantage of WGBS is that it can show the context of methylation, and, more importantly, absolute DNA methylation levels can be determined [2]. However, at the same time, WGBS is the most expensive and resource-demanding technique, since it requires a comprehensive genome coverage [2, 5–7, 30]. A way to reduce the cost of the experiment is to use RRBS, which is a popular choice when certain regions are of interest, rather than the whole genome [2, 7]. In RRBS, DNA is digested into short fragments with CpG dinucleotides at the ends using methylation-insensitive restriction enzyme *Msp*I, which recognizes 5′-CCGG-3′ sequences [7, 30]. Before bisulfite conversion and PCR, fragments that are rich in CpGs are selected—selection of 40–220-bp-long fragments has been shown to cover 85% of CGIs, mostly in promoter regions [30]. The disadvantage of RRBS is a low coverage of distal regulatory elements and intergenic regions, in which case WGBS would be a more appropriate choice [2]. However, it is important to note that bisulfite sequencing is not able to distinguish 5mC from 5hmC, because both modifications are resistant to conversion to uracil [11].

### DNA hydroxymethylation sequencing

There are several approaches designed specifically for 5hmC sequencing. The main idea behind oxidative bisulfite sequencing (**oxBS-seq**) is specific oxidation of 5hmC into 5fC or 5caC using potassium perruthenate [6, 11, 31]. 5fC and 5caC are converted to uracil nucleotides as unmethylated cytosines and oxBS-seq results in registration of 5mC excluding 5hmC. Therefore, a control BS-seq is necessary to recognize which positions were excluded in oxBS-seq [6, 11, 31]. Tet-assisted bisulfite sequencing (TAB-seq) comes as an improvement to the oxBS-seq, because it is able to read 5hmC directly [6, 11, 32]. 5hmC residues are protected by glucose moiety in the first step of the protocol [6, 32]. Unprotected 5mC nucleotides are then oxidized to 5fC or 5caC by TET and converted to uracil along with unmethylated cytosine. 5hmC are the only residues that are read as cytosines [6, 11, 32].

## Computational analysis of DNA methylation and hydroxymethylation sequencing data

The three main steps of computational analysis of DNA methylation data are as follow: (1) data processing and quality control; (2) data visualization and statistical analysis; (3) validation and interpretation [2, 3, 5]. For restriction enzyme- and enrichment affinity-based methods (MRE-seq, MeDIP-seq), data is analyzed by comparing the relative abundance of the fragments, while for bisulfite sequencing (WGBS and RRBS), methylation is called at individual cytosine residues and statistical testing for differential methylation can be performed by investigating DMPs or DMRs [2, 3].

### Bisulfite sequencing data processing

The processing of bisulfite sequencing data is challenging due to bisulfite conversion, which reduces sequence complexity significantly. C to T alignments are asymmetrical, i.e., Watson and Crick strands are not complementary, because conversion occurs only at Cs and not Gs [3, 5–7]. These are the main concerns that computational tools for bisulfite sequencing data analysis must be aware of and deal with, which is different compared with tools for regular DNA sequencing data analysis.

It is important to ensure a high quality of the sequencing reads in order to get a good alignment, and later correct methylation scores [3]. Therefore, prior to the alignment step incorrectly converted reads should be discarded and reads with adapter sequences must be found and adapters trimmed, using for example *Cutadapt* [5, 33]. Some pipelines have these features included already. Trimmed sequencing reads are aligned to the reference genome and methylation is called. Aligners for sequencing data are based on two types of algorithms: wild-card or three-letter [2, 3, 5, 34, 35]. Bisulfite aligners output aligned reads along with methylation calls for each C with sequence context information. The wild-card algorithm substitutes Cs with Ys (wildcards) in the reference genome, so reads can be aligned with both, Cs and Ts [3, 34, 35]. Examples of tools that integrate wild-card aligners are as follows: *LAST* [36], *BSMAP* [37], *RRBSMAP* [38], and *Pash* [39]. On the other hand, the three-letter algorithm converts all Cs into Ts, both in the reference genome and in the reads [3, 34, 35]. This reduces sequence complexity, but allows the adaptation of standard aligners, such as *Bowtie*. With three-letter aligners, many reads align to more than one position and are discarded, avoiding incorrect results, but DNA methylation information for some of the CpGs are lost [2, 3]. However, it is possible to align discarded reads to the best-matching positions to increase the coverage of CpGs [2, 3]. Examples of tools that integrate three-letter aligners: *Bismark* [40], *BRAT-BW* [41], *BS Seeker 3* [42].

Due to beforementioned issues related to asymmetrical alignments and non-complementarity, post-alignment tools are needed. It is possible to filter out the sites with best coverage, and also to calculate the average methylation levels and generate informative plots in order to see the scope of the problems in the alignments. Several tools can be used: *BSPAT* is able to summarize and visualize DNA methylation co-occurrence patterns and detect allele-specific methylation, while *SAAP-RRBS* can give the annotation of each C and report for high coverage and quality CpGs [2]. Another issue to be aware of during analysis is double counting of the same DNA fragments, which should be avoided by trimming overlapping parts of paired-end reads [3].

Finally, most of the tools and the principles of the computational analysis of DNA methylation data in this review are described for the more common single-base encoding sequencers (Illumina, Roche 454, Ion Torrent). If the data have been generated with a two-base encoding sequencer (ABI SOLiD), the bioinformatic pipeline is challenged and requires special attention [3].

### MRE-seq and MeDIP-seq data processing

For MRE-seq or MeDIP-seq, methylation levels are determined by comparing relative abundance of the fragments, i.e., the methylation information is in the enrichment or depletion of the sequencing reads [3]. Sequencing of the resulting libraries counts the frequency of specific DNA fragments in each library (methylated and unmethylated) and provides the raw data from which methylation levels can be inferred [3]. Unmethylated DNA can be enriched using unmethylated DNA-cutting enzymes (HELP-seq assay). A special attention should be put on handling the batch effect, which possibly can occur due to fluctuations in DNA sequencing coverage [3].

Processing of the MeDIP-seq data starts with the alignment, which is performed using standard aligners, such as *Bowtie* or *BWA* [3]. Relative enrichment scores are calculated by “extending the sequencing reads to the estimated DNA fragment size and counting the number of unique reads that overlap with each CpG or genomic regions of interest” [3]. However, methylation scores after alignment are confounded by an uneven CpG distribution in the genome, which must be corrected (normalized) [3, 43]. The most frequently described tools for data normalization are *BATMAN* and *MEDME* or the *MEDIPS* pipeline that combines both [3, 43]. *MeQA* and *MeDUSA* are the pipelines that include *BWA* and *MEDIPS*, making *MeDUSA* the most complete pipeline for MeDIP-seq data analysis [43]. Repitools is an R package that is recommended for quality control [3].

In the analysis of MRE-seq data, sequencing reads are aligned to the reference genome, using an all-purpose aligner of user’s choice. The restriction sites of the restriction enzyme used in the experiment must then be checked and matched [44]. DNA methylation is inferred by analysis of the read coverage. Differently from bisulfite sequencing and MeDIP-seq data analysis, just a few tools have been created specifically for MRE-seq data. One of them is the R package *msgbsR* [44].

### Data visualization and statistical analysis

Methylation data can be visualized in various genome browsers, such as *UCSC Genome Browser* or *Ensembl*, where the global distribution of a DNA methylation profile can be inspected [3]. Methylation tables in BED or bedGraph formats derived from data processing must be converted into bigBed or bigWig file formats that allow visualization of large data sets in genome browsers [3]. Various diagrams can be used to represent the data, including box plots, violin plots, tree-like diagrams, and scatter plots.

The next step after visualization is determination of DMPs and DMRs and this step is assay independent. Individual Cs are investigated and tested, but when differences are small, testing scale can be extended to a cluster of neighboring Cs [2, 7]. The actual size of a region can vary from a single C to entire gene loci, as it depends on the biological question and bioinformatic analysis pipeline. Most DMRs are between a few hundred to a few thousand bp long [3]. The difference between DMR detection in a bisulfite sequencing pipeline and enrichment-based sequencing pipeline is that for bisulfite sequencing, DMRs are detected from methylation tables, while for enrichment data they are detected directly from count data.

The success of DMP and DMR determination depends on computational power (which is especially important when genome-wide data is being analyzed) and the statistical testing itself [2]. The algorithms mostly adopt a sliding window approach across the genome to survey candidate DMRs [2]. Only the sites that are covered by the data from all samples are eligible for DMR testing. Statistical correction for multiple testing is essential, because many sites are tested simultaneously [2, 3]. The correction is done by controlling the false discovery rate (FDR) [3]. FDR inference for each DMR is often done using the *q value* method, but alternative approaches are also available [3]. Replicates help to improve the statistical significance of DMR detection; however, replicates are not always available for public data [2, 5]. Fisher’s exact test can be used when replicates are not available [7]. However, differences in DNA methylation may then be overestimated, because variation within groups is not taken into account [2]. Identified DMRs are ranked based on statistical significance and effect size (methylation difference, *t*-score from *t* test or *p* value). One of the most important tools for DMR detection is *BSmooth*, but *methylSig*, *methylPipe*, and *BiSeq* can also be used [2].

### Validation and interpretation of the differences

After the statistical analysis, the list of DMRs is generated and can be interpreted. It is useful to rank DMRs by *p* values, but relative and absolute differences in DNA methylation can be used as an additional measure for ranking [3]. The most highly ranked DMRs can be inspected manually in the genome browser. Often DMRs are validated in a new sample cohort and usually using locus-specific DNA methylation assays [3].

Interpretation of the DNA methylation and hydroxymethylation results depends highly on the experimental setup. However, computational tools can be helpful to explore the results. DMPs and DMRs are usually being associated with genes, based on their location relative to gene promoter or body. The now popular gene set enrichment analysis (GSEA) and pathway analysis can be performed for functional analysis, using web-based tools like *Enrichr* [45, 46] or *DAVID* [47]. GSEA aims to identify overrepresented genes and associate the set with possible phenotypes [48]. When DMRs are not mapped to specific genes, the *GREAT* (Genomic Regions Enrichment of Annotations Tool) approach can be used [49]. Correlating DNA methylation with gene expression patterns is a widely used strategy for interpretation of the functions and importance of the discovered DNA methylation changes. Moreover, DNA methylation data can be integrated with other omics data, such as ChIP-seq. New tools such as BioMethyl are emerging that integrate several algorithms specifically for interpretation of DNA methylation data [50]. However, interpretation of DNA methylation changes and omics data integration is still a challenging task, both technically and biologically, and should be an appropriate topic for a separate review in order to cover all relevant aspects.

## Specific computational tools for analyzing DNA methylation sequencing data

The following section describes several tools that have been developed to analyze DNA methylation sequencing data generated using the different experimental protocols presented above. For each experimental technique, we indicate which tool we believed to be the optimal choice for a scientist with limited knowledge in computational data analysis. For the selection and recommendations, we used criteria of performance (from the raw reads processing to differential analysis), graphic output options, and availability of a detailed manual (see Table 1). In addition, we took into account more practical criteria, such as how easy it was to download, install, and execute the particular tool, based on personal experience. The tools are recommended for each experimental protocol, according to the number of criteria that could be fulfilled. The recommendations are discussed in more detail under the “Discussion” section.

**Table 1:** Selected tools and their features. Whether the pipeline is capable of performing an analysis from raw reads to DMRs and DMPs was a crucial criterion for the selection of tools. However, other aspects, such as graphic output and availability of a detailed manual, were also important for the final recommendation

Selected tool	Experimental approach	From raw reads to DMPs and DMRs				Graphic output	Detailed manual available	Reference
		Quality control and 3′ trimming	Alignment	Methylation levels	Differential methylation			
*BSmooth*	WGBS	No	Yes	Yes	DMRs only	BED, bedGraph, Tab-del	Yes	[51]
*MOABS*	WGBS, RRBS, and possibly 5hmC seq	Yes	Yes	Yes, but no beta score	Yes	BED, bedGraph, Tab-del	Yes	[52]
*MethPipe*	WGBS and 5hmC seq	Error estimation only	Yes	Yes	Yes	BED, bedGraph, Tab-del	Yes	[53]
Bicycle*	WGBS and 5hmC seq	Yes	Yes	Yes	Yes	BED, bedGraph, Tab-del, VCF	Yes	[54]
SMAP*	RRBS only	Trimming only	Yes	Yes	Yes, also SNPs	Tab-del	Yes	[55]
*Genestack.com* (web-based)	WGBS and RRBS	Yes	Yes	Yes	No	Yes	Yes	[56]
*MeQA*	MeDIP-seq	Yes	Yes	Yes	No	No	Yes	[57]
MeDUSA*	MeDIP-seq	Yes	Yes	Yes	Yes	No	Yes	[43, 58]
msgbsR*	MRE-seq	Yes	No	Yes	Yes	Yes	Yes	[44, 59]

*Recommended

*Tab-del*, tab-delimited output

### Selected tools for bisulfite sequencing data analysis

Just a handful of tools can perform all or most of the necessary steps in the data analysis. For example, *BS Seeker*, *Bismark*, and *BSMAP* are suitable for bisulfite sequencing read alignment only [37, 40, 42], while *GBSA* and *BSmooth* are for specific downstream analyses [51, 60]. *BS Seeker* performs alignment and methylation calling, but does not calculate methylation ratio or beta scores [42]. On the other hand, *Bicycle* is able to perform all necessary steps and is relatively universal to different platforms [54], while *SMAP* is a great example of a convenient pipeline, but suitable only for RRBS data [55].

*BSmooth* is a tool for WGBS data analysis that performs alignment of the reads, measures methylation levels, and detects DMRs when biological replicates are available [51]. *BSmooth* takes into account biological variability (not only sample) while searching for DMRs. The algorithm detects regions consisting of several CpGs; thus, biologically significant differentially methylated single CpGs will be missed in the results, which can be a disadvantage in a research setting [51]. Working with the *BSmooth* algorithm can be challenging to many users, since data must be pre-processed and adapted for the analysis in an R environment. Considering the level of difficulty and the limited capabilities of the tool, it is therefore not recommended for most users (Table 1).

*MOABS* (Model-based Analysis of Bisulfite Sequencing data) is one of the most powerful command line-based pipelines that are suitable for WGBS, RRBS, and 5hmC data analysis [52]. It is able to perform alignments, methylation calling, identification of DMPs and DMRs, and differential methylation analysis (Table 1). It reports a unique value that combines biological and statistical significance for differential methylation—credible methylation difference (CDIF) [52]. Since the pipeline does not report beta score for methylation, it can be difficult to compare results from *MOABS* with results from other research projects. The *MOABS* pipeline offers powerful algorithms for data analysis. However, setup of the analysis is complicated and probably too complicated for users that are inexperienced with respect to command-line use. It seems to be complicated to organize the input and output files, and the user must be very familiar with writing definitions and paths. *MOABS* can be executed by writing a master/configuration script or by using command lines. Using a configuration script is more convenient, but the whole analysis is performed at once, which can be demanding regarding computational power and CPU time.

*MethPipe* is a pipeline similar to *MAOBS* and integrates various tools for methylation data analysis, including alignment, methylation calling, analysis of hypo- and hypermethylated regions, and differential methylation analysis (Table 1). It is also applicable for DNA hydroxymethylation analysis [53, 61]. However, *MethPipe* is considerably more difficult to use, compared with *Bicycle*, *SMAP*, or even *MOABS*, since it requires even more commands to be written and executed. On the other hand, writing and executing individual commands in the pipeline allows a maximum amount of control on the process: it can be run in small steps, with output files named and ordered according to user’s preferences. Furthermore, *MethPipe* has an extensive documentation with thorough instructions, which is useful to read even without intending to use the pipeline itself, since it describes the basic principles of DNA methylation data analysis [61]. *MethPipe* developers have also created and curate a reference methylome database *MethBase*, which can be useful for biological comparisons [53]. For example, by adding tracks of methylomes from different human tissues and cell lines to the UCSC browser and comparing them to own data. Data from *MethBase* can be downloaded using UCSC Table Browser or from the *MethBase* website for individual methylomes, where files contain methylation levels and coverage information for each CpG.

*MOABS* and *MethPipe* could be the pipelines of choice for more experienced users. However, because of its high functionality and user-friendly command line, *Bicycle* is the main pipeline we are suggesting for use by scientists with different backgrounds.

#### *Bicycle (recommended for WGBS, targeted BS-seq, and TAB-seq)*

*Bicycle* is a pipeline for computational analysis of bisulfite sequencing data that is more powerful or at least as powerful as *MOABS* or *MethPipe*, but undeniably easier to use, which is a great benefit for scientists without advanced computational skills [54]. The pipeline is able to perform all necessary steps—from conversion and indexing of the reference genome to the differential methylation analysis (Table 1). The tool is suitable for both paired-end and single-end reads. *Bicycle* has several advantages over other pipelines and includes more options than any other bisulfite sequencing analysis pipeline [54]. It can analyze the efficiency of the bisulfite conversion, which is important for correct estimation of methylation levels. Furthermore, it identifies and removes ambiguous reads, which is not included in other pipelines. Removal of clonal reads is also a *Bicycle* feature that is not often covered in methylation pipelines. No other pipeline has a non-CG to CG context correction option, while *Bicycle* performs it automatically during methylation analysis.

Methylation analysis of raw sequencing reads, and subsequent differential methylation analysis can be performed with just 4 commands, and 2 additional commands are required only when a reference genome is used for the first time [54].

The 6 steps in the pipeline (Additional file 1):

1. Creating a project. All output files are held in one folder.
2. Creating two in silico bisulfited reference genomes. C-to-T conversion for Watson strand reads and G-to-A conversion for Crick strand reads.
3. Indexing the reference genomes. Steps 2 and 3 are needed to be executed only for the reference when used for the first time.
4. Aligning the reads.
5. Methylation analysis and methylcytosine calling.
6. Determination of DMPs and DMRs. Differentially methylated positions are always determined, but when regions of interest are determined, only relevant positions alongside with differentially methylated regions are reported.

*Bicycle* creates two in silico bisulfited versions of reference genomes: C-to-T conversion is made to accommodate reads from the Watson strand and G-to-A conversion for the reads from the Crick strand [54]. Two versions of references are then indexed. Reads are processed concurrently and mapped to the references executing two separate threads. The mapping command outputs SAM files, which are then automatically converted to BAM files and indexed with *SAMtools* [54].

Each cytosine is visited and assigned to a methylation context (CG, CHG, or CHH). Methylation level calculation and methylation calling are performed [54]. Various corrections, which can be controlled by options, are performed automatically. For example, if a cytosine is initially assigned to CHG or CHH due to single-nucleotide polymorphism (SNP), it is re-assigned correctly to a CG. In this step, filters can be applied: disregard ambiguous reads, discard clonal reads and keeping the highest quality one, filter out incorrectly converted reads [54]. During methylation calling, at each position, bicycle estimates the error rate in bisulfite conversion by calculating the error as the percentage of unconverted Cs from an unmethylated control genome (when it is included in the experiment), by calculating the error as the percentage of unconverted barcodes (when barcodes with unmethylated Cs were attached to the reads before bisulfite conversion) or by using a specified fixed error rate [54].

The significant advantage of the *Bicycle* pipeline is that it also can perform a differential methylation analysis. Both DMPs and DMRs are computed by comparing to groups of samples (control and condition). The statistical analysis is based on MethylSig algorithm [62].

*Bicycle* can be adapted for the analysis of 5hmC, identified using the TAB-seq approach. 5hmC would be reported as methylated cytosine during the analysis with the pipeline. Analysis should be available for the oxBS-seq data as well, but then positions that overlap between oxBS-seq and BS-seq of the same sample should be discarded in order to identify 5hmC but leave 5mC modifications behind.

#### SMAP (recommended for RRBS)

*SMAP* is another example of a bisulfite sequencing data analysis pipeline [55]. It focuses on RRBS data analysis from reference preparation to detection of DMPs, DMRs, SNPs, and allele-specific methylation (ASM). In step 1 of the pipeline, the reference genome is prepared by converting all Cs into Ts for both strands and indexing those strands. Reference is cut into target regions, based on the enzyme that was used in the RRBS protocol. In step 2, reads are trimmed and aligned in step 3 (Additional file 1). Two alignment algorithms can be chosen: *Bowtie2* or *bsmap* and their options selected. In step 4, methylation levels are calculated for target regions. DMPs and DMRs are detected in step 5 using Fisher’s exact test when seed number is smaller than 5. Otherwise, *t* test or chi-square tests are chosen automatically. SNPs and ASM are analyzed in step 6 using *Bis-SNP* or *Bcftools*. Heterozygous SNPs are then filtered for ASM event detection. In a final step, results are summarized into a report [55].

### Web-based alternatives to command-line tools

There are several online pipelines for methylation analysis, where own data can be uploaded and analyzed using a visual interface rather than a command-line. However, often online platforms require frequent maintenance, and lack of this leads to poor website performance, annoying errors, and crashes. Another important concern is data protection for sensitive human genetic data in servers or clouds used by the particular platform, since data has to be uploaded to perform the analysis, and such data handling and storage is still a topic of discussion [63–65].

*Genestack.com* is an online platform that offers pipelines for the analysis of various data types, including WGBS (and RRBS) (https://genestack.com) [56]. A 30-day free trial is available in order to try the tools. However, since September 2019 access to the *Genestack* platform has been restricted and after the free trial period a paid subscription is required. The platform is visually pleasing and visualizes the results from all necessary steps of the methylation data analysis pipeline, which is a big advantage, compared with the command line-based tools. Unfortunately, big data upload is not efficient enough and is highly time-consuming. The advantage is that uploads can be resumed after some time even if computer is turned-off or internet connection interrupted. Furthermore, some of the available public data is already accessible and does not need to be uploaded. However, the access of the tools and their application to the data can be confusing, since they are not well listed in the menu. To make it easier, there is a task manager available to track the activity and access the results. In addition, the *Genestack* website has several thorough tutorials, created especially for the WGBS, RNA-Seq, and other omics data.

Mapping to the reference genome is performed using the *BSMAP* algorithm, and various options such as number of mismatches or the BS data generation protocol can be chosen. Unfortunately, differential methylation is not available in *Genestack*, which is a significant disadvantage of the platform. Overall, considering the disadvantages of the platform and controversies regarding the treatment of sensitive data, this platform would not be our first choice for data analysis (Table 1).

### MeDIP-seq data processing

The earliest tools developed specifically for MeDIP-seq data analysis were *Batman* and *MEDIPS* (which is possibly the most frequently used tool for MeDIP data analysis), but these tools do not perform quality control or mapping of the reads [57]. Therefore, additional tools are required to prepare the data for analysis, which is time-consuming and can be challenging computationally. As a solution, there are several pipelines that combine various tools, including *MEDIPS*. The most frequently described and recommended pipelines in various publications are *MeQA* and *MeDUSA*.

#### MeQA

Huang et al. created the *MeQA* pipeline for “pre-processing, data quality assessment and distribution of sequences reads, and estimation of DNA methylation levels of MeDIP-seq datasets” [57]. To run the pipeline, a configuration file must be prepared, which is then called by a command line. The pipeline consists of two main parts. Part A performs a quality control (summarized in a pdf report with graphs), and an alignment that results in BAM files and alignment quality control. Reference genome and indexes are downloaded automatically from UCSC, which is a great advantage of *MeQA*. DNA methylation levels are estimated in part B and mapped regions are extracted in BED format. The regions or parts of regions that correspond to promoters, bidirectional promoters, genes, or downstream of genes are identified and CpG enrichment is estimated. Summary of the results is generated.

Unfortunately, *MeQA* does not perform differential methylation analysis (DMR analysis) [57]. In addition, currently the pipeline seems to be unavailable, which prevents us from recommending it.

#### MeDUSA (recommended for MeDIP-seq data analysis)

*MeDUSA* (Methylated DNA Utility for Sequence Analysis) is a pipeline for MeDIP-seq data analysis that focuses on accurate DMRs detection [43, 58]. It contains several packages to perform a complete analysis of MeDIP-seq data: sequence alignment, quality control, and DMR identification (Table 1) [58]. *BWA* is used for the alignment, *SAMtools* for subsequent filtering, and *FastQC* for quality control metrics. *MeDUSA* integrates and uses *MEDIPS* as a tool for methylation analysis. The pipeline is executed by writing a configuration file, which runs the scripts of the pipeline. Template and example configuration files are available to download.

The pipeline consists of four parts. In part 1, the alignment of reads and filtering is performed, using *BWA* and *SAMtools*. Some of the alignment parameters are set up in the configuration file, while more can be added by modifying the part 1 script. The part 2 script runs *MEDIPS* and its quality control and generates WIG tracks for individual strands and both strands combined. The tracks are converted to bigWig format. DMRs are called in part 3 using *MEDIPS*. In part 4, these DMRs are annotated (Additional file 1). In this step, annotation files are required, and they must be written in GFF file format and organized in the correct directory structure. Annotation files are available together with *MeDUSA v2.0*, while the newest version *2.1* does not include these files. However, they can easily be copied from one version to another.

### MRE-seq data processing

MRE-seq is not the most popular approach to study DNA methylation, although some datasets are publicly available and have potential to be used. Therefore, developing specific tools and pipelines for this type of data is not common. However, R Bioconductor has a package just for methylation-sensitive restriction enzyme sequencing data, *msgbsR* [44].

#### msgbsR (recommended for MRE-seq data analysis)

The methylation sensitive genotyping by sequencing R package (*msgbsR*) contains a collection of functions for MRE-seq data analysis [44, 59]. However, the input must be indexed BAM files, which means that the user must do data pre-processing before using *msgbsR*. This can be done with *Bowtie2* or *BWA* aligners. msgbsR then identifies and quantifies read counts at methylated sites. Enzyme cut sites are also verified and DNA methylation is assessed based on read coverage [44]. One of the advantages of this package is the differential methylation analysis.

In the pipeline, the input BAM files are read. Then cut sites are extracted and checked. Incorrect cuts are filtered out and a preliminary read count table is generated. *msgbsR* can plot the results using *plotCounts*.

The user should keep in mind that this package requires pre-processing of raw data and knowledge of the R programming language and analyzing MRE-seq data means that both the R programming language and command-line tools will have to be used. However, an example script is provided on the website together with a manual [59].

## Discussion

As shown above, there is a large variety of tools available for analyzing DNA methylation sequencing data. The analysis of such data includes several main steps that each consists of several more detailed procedures. However, the majority of existing reviews only lists and recommends tools for individual steps [2–6]. Consequently, for a researcher, who is not sufficiently experienced in big data analysis and bioinformatics, finding and choosing the most appropriate tools for an analysis can be rather overwhelming. Furthermore, most of the tools are command line-based with complicated installation and execution procedures, and therefore challenging to integrate in the analysis workflow, especially when input and output files need to be converted to fit the particular tool. Often detailed instructions for this are not available. At the same time, beginners in DNA methylation sequencing data analysis must obviously learn the details of the chosen tools or pipelines in order to perform and understand the analysis in a reliable way, and we hope that this review can be a good starting point for initiating such analyses and provide relevant references. Here we have described available tools and pipelines, discussed their main advantages and disadvantages, and suggested a few that in our opinion currently represent the most optimal choice for DNA methylation sequencing data analysis. In the review, we also point out the need for pipelines that are powerful enough to fully analyze DNA methylation sequencing data—from raw sequencing reads to differentially methylated positions and/or regions (Table 1). Some of the pipelines can also be adapted to DNA hydroxymethylation data analysis.

*Bicycle* is the pipeline we have chosen to feature the most. Even though it is a command line-based pipeline, it is described so well that downloading and running the software is relatively straightforward [54]. It is important to understand that for big data analysis, such as DNA methylation, using command-line tools is the most convenient way to perform the analysis, since publicly available raw data are more easily downloaded and processed by using command-line tools. To enable effective analysis, pipelines should not be too complicated to use. Advantages of *Bicycle* are its simplicity, yet universality and functionality. The pipeline is suitable for bisulfite sequencing analysis and can also be adapted to DNA hydroxymethylation data analysis. It performs all essential steps and has more additional options than many other pipelines [54], like identification of ambiguous reads or beta score calculation. The options can easily be specified in commands, compared with more complicated command lines in *MethPipe* or *MOABS*, where a sophisticated configuration file is required [52, 53]. Probably the biggest drawback of the *Bicycle* (and in fact, many other command-line based pipelines) is a lack of graphic output, because it only provides VCF file for possible visualization in UCSC genome browser, whereas results from differential methylation analysis are in text files only.

Unfortunately, *Bicycle* does not include a workflow for RRBS data analysis, which could be a suggestion for a potential pipeline improvement in the future, thereby making the tool truly universal for bisulfite sequencing. *SMAP* is an optimal pipeline example to fill the missing gap of RRBS data analysis. Like *Bicycle*, it performs all necessary steps in the analysis and outputs DMPs, DMRs and, if required, SNPs and allele-specific methylations [55]. Even though *SMAP* is run by writing a configuration file, example files and user manual make it easy enough to use for an unexperienced user.

One way to avoid command-line tools would be to use online platforms. Arguably the biggest advantage of such platforms is a user-friendly interface, where analysis is performed by clicking and/or dragging actions. Furthermore, these platforms usually output attractive graphs that almost instantly can be used in reports and papers, while after *Bicycle* or *SMAP* analysis such graphs would need to be generated separately using sometimes complicated software. However, online platforms entirely depend on the servers and developers, who must maintain the service to keep it active. In addition, since the whole analysis of DNA methylation is a time-consuming process, web page crashes are a significant risk. *Genestack* is an online platform that has a solution for this problem by saving the progress of analysis and even the file uploads. Nevertheless, comparing functionality and accessibility of command line-based tools and online platforms, command-line should still be a first choice for big data analysis.

With respect to DNA hydroxymethylation analysis, there are to our knowledge no tools exclusively for this type of data, but many pipelines for WGBS analysis could be adapted (for example, *Bicycle* pipeline). However, for some other tools, possible adaptations must be assumed by the user, since documentation is not extensive enough or recommendations are not clear. A lot is fundamentally unknown about hydroxymethylation, so development of more tools targeting this specific DNA modification should be the focus of future developments.

Enrichment and restriction-based approaches for DNA methylation analysis are not as popular today as WGBS, RRBS, or microarrays. Consequently, there are fewer available tools and pipelines for this type of data, and few new tools are being developed. Nevertheless, MRE-seq and especially MeDIP-seq are still used, when single-nucleotide resolution is not needed, and numerous datasets produced with these techniques are publicly available. *MeDUSA* is a pipeline we recommend for MeDIP-seq analysis. The pipeline integrates *MEDIPS* for indicating the robustness of the normalization algorithm. This might mean that the tools for MeDIP-seq analysis are efficient enough to eliminate the need for a big variety of tools. For example, the *MeQA* pipeline is currently unavailable to download, leaving *MeDUSA* the main pipeline for MeDIP-seq data analysis.

Third-generation sequencing approaches, including single-cell sequencing, nanopore sequencing, and single-molecule real-time sequencing (SMRT) technologies, have been created and already adapted to DNA methylation detection [2]. The wider use of new experimental approaches will eventually lead to a larger number of such datasets becoming available in databases, thus creating a need for user-friendly tools and pipelines created specifically for this type of data analysis. Third-generation DNA methylation data analysis is therefore an important direction for future discussions, but for the time being, the methods described in this review still dominate.

Recommendations for tools and pipelines are very important for scientists, especially according to their level of difficulty and requirements regarding computational experience. After reviewing the possibilities for DNA methylation sequencing analysis, it can be concluded that the current landscape of available pipelines and tools, especially for bisulfite sequencing data analysis, appears chaotic and without specific recommendations. A majority of the tools are suitable only for individual steps, or a selection of steps. Existing pipelines are mostly incomplete or still not widely used, often due to lack of maintenance. However, if users give proper credit to the creators of pipelines, including comprehensive citations, we believe that this would lead to fruitful discussions inside the user community, drawing attention to these pipelines and tools as well as their creators. This may improve the chance that a tool stays available in the future and is kept constantly improved and updated. It is crucial to properly describe the methods that were used in research projects to process different types of data. When it comes to the Methods section, researchers should be more detailed in describing computational and statistical data analysis and properly include the references and name the tools and algorithms that were used, and carefully define how they were combined into a workflow. Researchers could take example from Methods sections for experimental procedures, where over the time scientists have learned to meticulously list all materials and define exactly how they were used. In computational projects, often not a single, but a series of individual steps (using a selection of tools or even parts of different algorithms) are used to achieve the result. If these steps are clearly defined, then it becomes easier to understand the computational process of the research project and subsequently reproduce the results, which should be a sign of high-quality science.

## Conclusions

*Bicycle* is the main recommended pipeline for the whole-genome bisulfite sequencing and targeted bisulfite sequencing data analysis. This pipeline fits the criteria of being universal, highly functional, but at the same time easy enough even for the scientists with no or limited experience with computational analysis. Some pipelines, including *Bicycle*, can be adapted to DNA hydroxymethylation data analysis, but there are no specific tools yet for this type of data. Another tool, *SMAP*, is recommended specifically for reduced-representation bisulfite data analysis, while *MeDUSA* is a pipeline of choice for analyzing precipitated methylated DNA. Recommended pipelines were picked from a large variety of available tools, where some are described better than others. Some are also used more often, and hence they are being updated more regularly, while others are no longer available. It is important to acknowledge computational tools and pipelines in scientific papers, and to communicate with the authors, which could lead to more regular updates, improved manuals, and better selection guidelines.

## Supplementary information

**Additional file 1.** The file consists of short instructions on how to run recommended tools and pipelines, including some examples.

## Data Availability

Not applicable

## Abbreviations

5caC: 5-Carboxylcytosine
5fC: 5-Formylcytosine
5hmC: 5-Hydroxymethylation
5mC: 5-Methylcytosine
ASM: Allele-specific methylation
BS-seq: Bisulfite sequencing
CDIF: Credible methylation difference
CGI: CpG island
DMP: Differentially methylated position
DMR: Differentially methylated regions
DNMTs: DNA methyltransferases
FDR: False discovery rate
GSEA: Gene set enrichment analysis
hMeDIP: Hydroxymethylated DNA immunoprecipitation
MBD: Methyl-binding domain
MBDCap-seq: Methyl-binding domain-capture sequencing
MeCP2: Methyl-CpG-binding protein 2
MeDIP: Methylated DNA immunoprecipitation
MethylCap-seq: Methyl-capture sequencing
MRE: Methylation-sensitive restriction enzyme
OxBS-seq: Oxidative bisulfite sequencing
RRBS: Reduced-representation bisulfite sequencing
SAM: S-Adenosyl-L-methionine
SNP: Single-nucleotide polymorphism
TAB-seq: Tet-assisted bisulfite sequencing
TET proteins: Ten-eleven translocation proteins
TSS: Transcription start site
WGBS: Whole-genome bisulfite sequencing
