# Supplementary material for: DNA methylation data by sequencing: experimental approaches and recommendations for tools and pipelines for data analysis
Source: Clin Epigenetics. 2019 Dec 12;11:193. doi: 10.1186/s13148-019-0795-x (PMC6909609; doi:10.1186/s13148-019-0795-x)
Supplement: Supplementary file 1 — Additional file 1. The file consists of short instructions on how to run recommended tools and pipelines, including some examples. [file 13148_2019_795_MOESM1_ESM.pdf]

DNA METHYLATION DATA BY SEQUENCING: EXPERIMENTAL APPROACHES AND RECOMMENDATIONS FOR TOOLS AND PIPELINES FOR DATA ANALYSIS

Ieva Rauluseviciute[1*], Finn Drabløs[1], Morten Beck Rye[1,2]

[1] Department of Clinical and Molecular Medicine, NTNU - Norwegian University of Science and Technology, NO-7491 Trondheim, Norway

[2] Clinic of Surgery, St. Olavs Hospital, Trondheim University Hospital, NO-7030 Trondheim, Norway

* Corresponding author:

Ieva Rauluseviciute
Department of Clinical and Molecular Medicine
Norwegian University of Science and Technology
P.O. Box 8905
NO-7491 Trondheim
Norway
Email: ieva.rauluseviciute@gmail.com
Phone: +4796707578

How to run WGBS data analysis using Bicycle?

Requirements for Bicycle

Bicycle requires *Java* (1.8 or higher), *Bowtie* (0.12.7 to 1.0.0) or *Bowtie2* (2.3.2 or higher) and *SAMtools* (0.1.8 or higher) to be installed, but knowledge of the usage of these algorithms is not necessary. Reference genome must be in FASTA format, while raw sample data in FASTAQ. If replicates are available, they can be pooled to increase the coverage.

Data

Whole genome bisulfite sequencing data from prostate cancer (PCa) and prostate benign samples was chosen for the analysis. GEO access number GSE104789. Data was produced using Illumina HiSeq 2500 platform and contains 11 PCa and 3 benign tissue samples. Paired-end data from 2 PCa and 2 benign samples was downloaded and analyzed Table 1.

Table 1 **Information about samples used in data analysis.**

DNA methylation GSE104789				
**Run**	***Further alias after downsampling***	***Cancer/Benign***	***Spots***	***Bases***
**SRR6156048**	Met1	Cancer	259.8 M	36.5 G
**SRR6156046**	Met 2	Cancer	208.2 M	29.1 G
**SRR6156037**	Men 1	Benign	222.5 M	31.2 G
**SRR6156036**	Men 2	Benign	268.1 M	37.6 G

To download SRA files from GEO command `prefetch` from SRA tools is used:

```
prefetch -v -O /local/home/username/analysis/data/ SRR6156048
```

SRA files are converted to FASTAQ file format and splitted into two files with forward and reverse reads (_1.fastq is forward reads and _2.fastq is reverse reads) with `fastq-dump --split-3`:

```
fastq-dump --split-3 SRR6156048.sra
```

Two commands are repeated for four samples. In case dataset has technical replicates, data from them can be pooled using `cat`. In a presented case there were no replicates available. Example of pooling:

```
cat /local/home/username/analysis/data/replicate1.fastq
/local/home/username/analysis/data/replicate2.fastq
/local/home/username/analysis/data/replicate3.fastq >
/local/home/username/analysis/data/pooledreads.fastq
```

Sequences for all four samples were downsampled by 95% in order to fasten up the analysis. The new aliases for samples were introduced (Table 1).

Analysis with Bicycle

STEP 1. Creating a project

```
bicycle create-project -p analysis/project -r
analysis/referenceGenome -f analysis/data -m _1.fastq
```

It is essential that samples are organized in separate folders — one folder per sample.
*-p: project directory (`analysis/project`)
*-r: reference directory (`analysis/referenceGenome`)
*-f: reads directory (`analysis/data`)
* - required options
-b: bowtie directory (if not in PATH)
-b2: bowtie2 directory (if not in PATH)
-s: samtools directory (if not in PATH)
-n: bs-seq was made in non-directional protocol
-m: enable paired-end mode (regular expression)

STEP 2. Creating Watson and Crick in-silico bisulfited reference genomes

[Step is required for a newly downloaded reference genome]

```
bicycle reference-bisulfitation -p analysis/project
```

*-p: project directory (`analysis/project`)

STEP 3. Indexing the reference genomes

[Step is required for a newly downloaded reference genome]

```
bicycle reference-index -p analysis/project
```

*-p: project directory (`analysis/project`)

-v: bowtie versions to be used (1 or 2). Default is 2.

-t: number of treads (for bowtie2 only). Default is 2.

STEP 4. Aligning reads to the references

```
bicycle align -p analysis/project -t 4 -q phred33
```

*-p: project directory (`analysis/project`)

Some possible options:

-t: number of threads. Default is 4.

-e: maximum permitted total of quality values at all mismatched read positions throughout the entire alignment. Default is 140.

-l: seed length. The lowest permitted setting is 5. Bowtie is faster for larger values of -l. Default is 20.

-n: maximum number of mismatches permitted in the "seed". This may be 0, 1, 2 or 3. Default is 0.

-I: minimum insert size for valid paired-end alignments (paired-end projects only). Default is 0.

-X: maximum insert size for valid paired-end alignments (paired-end projects only). Default is 250.

-q: how qualities will be treated. Valid values are: solexa1.3, solexa, phred33, phred64, integer. Default is solexa1.3.

Phred scale should be determined according to the platform used to sequence. Phred quality score is a measure of the quality of the identification of the nucleobases [36, 37]. Fastq format contains phred scores along the read sequences. Phred scales and corresponding platform:

- Phred+33 for Sanger and Illumina 1.8 or higher.
- Solexa for Solexa.
- Phred+64 for Illumina 1.3 and Illumina 1.5.

To make sure, which phred value should be used in the command, fastq read files can be checked and based on the table (https://data.bits.vib.be/pub/trainingen/cheat-sheets/phred_to_probability.pdf) phred scale can be determined.

Alignment statistics are placed in .log files. One group of files are for WATSON reference and one for CRICK. To know the success of the alignment and other metrics open any one of the .log files, except head.log.

STEP 5. Methylation analysis and methylcytosine calling

```
bicycle analyze-methylation -p analysis/project -t 4 -a -o
```

*-p: project directory (`analysis/project`)

-n: number of threads. Default is 4.

-r: ignore non-correctly bisulfite-converted reads

-a: ignore reads aligned to both Watson and Crick strands

-o: ignore reads with more than one possible alignment

-t: trim reads to the <t> mismatch. 0 means no trim. Default is 4.

-d: ignore positions with less than <d> reads. Default is 1.

-f: FDR threshold. Default is 0.01.

-e: error rate computation mode. Options are:
from_control_genome=<control_genome_name>, from_barcodes or
FIXED=<watson_error_rate,crick_error_rate>. Default is FIXED=0.01,0.01.

-b: comma-separated (no spaces) list of BED files to annotate cytosines.

-c: remove clonal reads.

-g: correct non-CG.

Explanations on output files in this step:

- ○ **met1_hg19.fa.summary**. Methylation analysis results (error computation results, p-value cutoffs). One file per sample.
- ○ **met1_hg19.fa.methylcytosines**. A line for each cytosine in the reference with methylation level and FDR-adjusted significance value reported.
- ○ **met1_hg19.fa.methylcytosines.vcf**. Methylation levels for each C again, but VCF can be used to visualize results in UCSC.

STEP 6. Analyzing differential methylation

```
bicycle analyze-differential-methylation -p analysis/project -c
men1,men2 -t met1,met2
```

*-p: project directory (`analysis/project`)
*-t: comma-separated (with no spaces) list of sample names belonging to 'treatment' group
*-c: comma-separated (with no spaces) list of sample names belonging to 'control' group
-x: comma-separated (with no spaces) list of CpG contexts to analyze: CG, CHG or CHH. For example: CG,CHG. Default is CG.
-b: comma-separated (with no spaces) list of BED files to analyze at region-level.

Explanations on output files in this step:

- ○ **hg19.fa_met1_met2__VS___men1_men2.DMC.tsv**. Report of differential methylation for each cytosine (chromosome and cytosine position, methylation context, cytosine methylation for each sample of the treatment and control, methylation average for each condition, fold-change of treatment/control methylation in log2, p-value and q-value).
- ○ (reference_control__VS___treatment.bed-file.DMR.tsv. If BED file with regions of interest is determined, this file reports differential methylation for each annotated region.)

How to run RRBS data analysis with SMAP?

The pipeline is executed by modifying and running the configuration script, which is simpler and more straightforward, compared with, for example, the *MOABS* pipeline.

Firstly, the analysis needs to be configured and path of the output files determined:

```
perl ./Monitor.pl -c configure -o [Output path]
```

SMAP configuration file includes variables, paths of external software, data and sample files. The example of configuration file, where main settings need to be determined:

```
# A) Variables
# Working mode: multicore, sge, slurm
Mode = multicore
# Breakpoint switch: on, off
Bpt = off
# Sge queue (qsub command arguments "-q")
SgeQueue = bc.q
# Sge project (qsub command arguments "-p")
SgeProj = HUMcccR
# Slurm partition (sbatch command arguments "-p")
SlurmPart = test

# B) External Software
# Software for alignment (bowtie2 or bsmap)
Alignsoft = bsmap -z 64 -p 12 -s 12 -v 10 -q 2 -m 0 -x 800
# Absolute full path for java and R
Javasoft = /usr/bin/java
Rscript = /usr/bin/R

# C) Data files
# Reference
Reference = ./hg19/hg19.fa
# Anno dir
Annodir = ./data/element/
```

```
# Adapter file name
Adapter = NULL
Adapter = ./data/sample/adapter.fa
# Region
Region = ./data/common/sRRBS_40-300_region.bed
# Target
Target = ./data/common/Target.number

# D) Sample files
# Name of tissue, Normal or Tumor, single-end (SE) or paired-end (PE), name of the library, name of FlowCell, file name of read1, file name of read2, platform, target region (if needed).
Sample = Tissue Normal PE Library FlowCell ./data/sample/normal.1.fq ./data/sample/normal.2.fq illumina
Sample = Tissue Tumor PE Library FlowCell ./data/sample/tumor.1.fq ./data/sample/tumor.2.fq illumina
```

After configuration, analysis script (`sh RRBS_Run.sh`) is generated and program has to be run from the output directory:

```
cd /local/home/username/smap_analysis
```

Running script command:

```
sh RRBS_Run.sh
```

How to run MEDIP-seq data analysis with MeDUSA?

MeDUSA pipeline is executed by writing a configuration file, which then runs the scripts of the pipeline. Template and example of a configuration file are provided with a download package.

In the configuration file various paths must be determined: paths to pipeline scripts, read data, reference genome directory etc. In addition, paths to annotation files can be added. These files are provided for mouse and human, but own annotation files can also be added. Then information about reads — maximum insert length and read length — must be stated. Finally, thresholds for calling of differentially methylated regions must be determined. It includes minimum read depth, window size, DMR size and DMR *p*-value threshold. Default values are 10, 100, 500 and 0.1, respectively. Additional thresholds for annotation are available: intersect threshold (when a particular number of DMRs are overlapping with a feature, it is checked if that feature overlaps with the DMR), upstream and downstream nearest genes thresholds.

Executing the pipeline, it is necessary to determine the configuration file name and state which samples are control and which ones are treatment samples. If control samples are not available, this option can be turned off. The main command of the pipeline:

```
perl medusa.pl -p medusa_example.cfg -t disease_A,disease_B -c
control_A,control_B &
```

*-p: MeDUSA configuration file (`medusa_example.cfg`)
*-t: list of ids for treatment samples (`disease_A,disease_B`)
*-c: list of ids for control samples (`control_A,control_B`). If it is working with a single cohort option -c can be turned off by writing “0”.
-h: list options
* - required options
